# Indexing Mental Workload During Simulated Air Traffic Control Tasks by Means of Dual Frequency Head Maps

**DOI:** 10.3389/fphys.2020.00300

**Published:** 2020-04-21

**Authors:** Thea Radüntz, Norbert Fürstenau, Thorsten Mühlhausen, Beate Meffert

**Affiliations:** ^1^Mental Health and Cognitive Capacity, Work and Health, Federal Institute for Occupational Safety and Health, Berlin, Germany; ^2^Institute of Flight Guidance, German Aerospace Center, Braunschweig, Germany; ^3^Signal Processing and Pattern Recognition, Department of Computer Science, Humboldt-Universität zu Berlin, Berlin, Germany

**Keywords:** mental workload, psychophysiology, air traffic controllers, electroencephalography, biomedical signal processing, pattern recognition, state monitoring

## Abstract

In our digitized society, advanced information and communication technology and highly interactive work environments impose high demands on cognitive capacity. Optimal workload conditions are important for assuring employee's health and safety of other persons. This is particularly relevant in safety-critical occupations, such as air traffic control. For measuring mental workload using the EEG, we have developed the method of Dual Frequency Head Maps (DFHM). The method was tested and validated already under laboratory conditions. However, validation of the method regarding reliability and reproducibility of results under realistic settings and real world scenarios was still required. In our study, we examined 21 air traffic controllers during arrival management tasks. Mental workload variations were achieved by simulation scenarios with different number of aircraft and the occurrence of a priority-flight request as an exceptional event. The workload was assessed using the EEG-based DFHM-workload index and instantaneous self-assessment questionnaire. The DFHM-workload index gave stable results with highly significant correlations between scenarios with similar traffic-load conditions (*r* between 0.671 and 0.809, *p* ≤ 0.001). For subjects reporting that they experienced workload variation between the different scenarios, the DFHM-workload index yielded significant differences between traffic-load levels and priority-flight request conditions. For subjects who did not report to experience workload variations between the scenarios, the DFHM-workload index did not yield any significant differences for any of the factors. We currently conclude that the DFHM-workload index reveals potential for applications outside the laboratory and yields stable results without retraining of the classifiers neither regarding new subjects nor new tasks.

## 1. Introduction

In our digitized society, advanced information and communication technology and highly interactive work environments impose high demands on cognitive capacity and on the ability to cope with increased task load (Kompier and Kristensen, 2001; Niosh, 2002; Landsbergis et al., 2003; Lohmann-Haislah, 2012). According to several authors mental workload can be conceived as the amount of cognitive demands required in order to solve a task related to the cognitive resources available (Kahneman, 1973; Eggemeier et al., 1991; Xie and Salvendy, 2000; Wickens, 2002).

Optimal workload conditions are important for the health of the single individual and in order to assure the safety of other persons. Latter is particularly relevant in safety-critical occupations with high cognitive demands and responsibility, such as air traffic control. A valid and reliable method for measuring mental workload would offer a way for achieving such conditions in human-machine systems by capturing the instantaneous workload continuously over time (Byrne and Parasuraman, 1996; Scerbo et al., 2001; Arico et al., 2017). It is important that the registration method does not interact with the task or alter subject's mental state by imposing additional demands as it is the case during subjective assessment of workload by means of questionnaires. Furthermore, the workload should not only be detectable in retrospect or after the occurrence of errors as it is the case when performance measures are used for workload detection. Thus, questionnaires and performance evaluation are only of limited relevance for real-time analysis of workload conditions in the range of seconds.

Over the past 50 years, various physiological parameters (e.g., heart rate and derived parameters, electrodermal activity, body temperature, etc.) have been evaluated for their validity regarding continuous mental workload registration. Since the discovery of the electroencephalogram (EEG) by Berger (1929), relations between bioelectric brain activity and cognitive states have been studied. Improvements of the amplifier technology and computerized evaluation of biosignals made systematic investigations possible. In last century's 90s, the state-of-the art regarding EEG's evaluation and validity was summarized in reviews that served as a starting point for the use of the EEG in applied research, e.g., in human-factors. In a review article, Borghini et al. (2014) provided a detailed overview of the measurement of neurophysiological signals for the determination of mental workload and confirmed essentially the known relations. The authors further concluded that no convincing algorithms were available for a reliable online workload detection.

The spectral power of oscillations in different frequency bands were used as parameters for describing the spontaneous brain activity. For the alpha-frequency (8–12 Hz) and theta-frequency (4–8 Hz) bands, spectral-power comparisons in all relevant investigations described systematic relations to cognitive and memory performance (Sterman and Mann, 1995; Pfurtscheller, 1997; Gevins et al., 1998; Klimesch, 1999; Gevins and Smith, 2000). These EEG bands were also linked to different levels of workload by means of analysis of variance (e.g., Mecklinger et al., 1992; McEvoy et al., 2001; Lei and Roetting, 2011; Brouwer et al., 2012; Capilla et al., 2012; Aricò et al., 2018) and demonstrated a decrease of the alpha-frequency band power and an increase of the theta-frequency band power with increasing mental workload.

In recent years, however, classifiers were increasingly used for the separation of workload levels. The feature vectors—derived from the EEG—revealed varying complexity and extent, and frequency bands were taken differently into account. The used EEG parameters were, for example, the amplitude of the EEG signal, spectral power of different frequency bands, and different EEG channels (Wilson and Russell, 2003b; Lin et al., 2006; Kohlmorgen et al., 2007; Baldwin and Penaranda, 2012; Penaranda and Baldwin, 2012; Ke et al., 2014). The focus was on frontal, parietal and occipital EEG channels according to previous findings. Independent component analysis (ICA) was used to determine specific reactions of spatio-temporal different sources (Gardony et al., 2017) and allowed the successful detection and elimination of artifacts (Mognon et al., 2011; Radüntz et al., 2017; Puma et al., 2018).

Initially, studies that dealt with the determination of workload were conducted in the laboratory using different task batteries (Gevins et al., 1998; Gevins and Smith, 2000; McEvoy et al., 2001; Berka et al., 2007; Grimes et al., 2008; Baldwin and Penaranda, 2012; Brouwer et al., 2012, 2014; Christensen and Estepp, 2013; Weiland et al., 2013; Gerjets et al., 2014; Hogervorst et al., 2014; Ke et al., 2014; Hou et al., 2016; Gardony et al., 2017; Rosen and Reiner, 2017; Puma et al., 2018). Meanwhile, investigations of cognitive workload with more realistic tasks became more popular (Kohlmorgen et al., 2007; Lei and Roetting, 2011; Aricò et al., 2018; Dehais et al., 2018). Air traffic controllers (ATCOs) pose a special challenge due to the complex task-load situations with changing activities and strategies for air traffic management (ATM). The requirements can change very fast, a clear and direct objective graduation of task-load proves to be difficult, and the transitions are often unpredictable and fast. Experiments with ATM simulations and a task-load grading proved to be advantageous although the majority of simulated ATM examinations were limited to two task-load levels (easy and difficult). Relevant studies on workload determination methods for simulated or real air traffic control were conducted by Brookings et al. (1996), Wilson and Russell (2003b, 2007), Shou et al. (2012), Abbass et al. (2014b,c), Borghini et al. (2014, 2017), Aricò et al. (2015, 2016); Aricò et al. (2018), Di Flumeri et al. (2015), Dasari et al. (2017), and Dehais et al. (2018).

Wilson and Russell (2003a) investigated the classification of the mental state of seven air traffic controllers in simulated air traffic monitoring. In seven different task-load conditions a 19-channel EEG, heart rate, blink rate, and respiratory rate were recorded. The spectral power of five frequency bands was calculated for each EEG channel from 1-s windows and used per subject as input for the artificial neuronal networks (ANN) and stepwise linear discriminant analysis (SWLDA). Discrimination only between two conditions yielded the best result with an accuracy of 97.5% (ANN) and 91% (SWLDA). Thereby only 22 relevant features were included in the evaluation. The authors drew attention to the following open questions of day-to-day variability of psychophysiological measures and long training duration for ANN. They stated that a one-size-fits-all solution would be beneficial.

Abbass et al. (2014c) dealt with questions about visual and auditory information processing in relation to mental workload of air traffic controllers. In addition, the authors examined the question of whether a narrow-band frequency resolution of the EEG was better suited for the assessment of workload. They found that there were no quantitative advantages over the usual frequency bands. Further, they suggested to focus on the separation of high and low workload and neglect the middle range (Abbass et al., 2014a).

The question of reliability of EEG-based workload determination in ATM tasks was examined in Arico et al. (2015). According to the authors the reason for the decreasing classification accuracy over days, as reported by Christensen and Estepp (2013), could be overfitting, i.e., a too high specificity of the training data. It was hypothesized that a simple classifier based on fewer spectral properties guaranteed a high selectivity over days. Twelve ATCO interns completed the simulated ATM task on 2 consecutive days and after 9 days. The EEG was registered by 13-channels and 2 s windows were used to compute relevant EEG spectral features. For each subject, cross-validation of the classifier between the days was calculated using 5, 50, and 100% of relevant EEG features. The results showed that the use of only 5% of the relevant features contributed to an over-day stable workload measurement.

Basically, changes in the alpha-frequency and theta-frequency band powers related to mental workload have been confirmed many times and proved to be meaningful in accordance with the findings of the last 50 years. The majority of workload studies dealt with the analysis of the EEG during cognitive tasks related to working memory and executive control. While some authors investigated whether a brain-state monitoring was possible on the basis of universal and general activation signs in the EEG (Bashivan et al., 2014, 2015; Ke et al., 2014), others tested the possibilities and limitations of over task requirements (cross-task training) and inter-individually (cross-subject training) transferable classifiers. Discrimination accuracy of the classifiers between high and low workload was often not sufficient in cross-task training and remained below the significance threshold. Cross-subject training of the classifier was also less favorable than intra-subject classification. In the driving simulator study by Kohlmorgen et al. (2007), the authors concluded that a highly adaptive approach was needed to account for the neurophysiological variations. According to the authors, a universally applicable “workload detector” with fixed parameters did not seem to be realistic at the moment. The selection of appropriate data for classifier's training needs more elucidation. This is especially important as frequent allegations were made concerning the time interval between training and test of the classifier that proved to be particular relevant for the classification accuracy (Penaranda and Baldwin, 2012). In order to avoid overfitting and increase the stability of the classifier performance over time a smaller number of features could be beneficial (Arico et al., 2015).

It has to be stated that different cognitive strategies in task solving, both intra- and inter-individually, can influence the classification results. In this context, Puma et al. (2018) suggested to cluster the subjects according to their performance, age (McEvoy et al., 2001), and individual experiences. These should be considered if workload registration methods are to be validated.

Based on the possibility that machine learning algorithms provide the ability of workload registration in the range of seconds, the question arises whether they provide reliable and reproducible results over time, in particular without the need for re-training of the classifier regarding subjects and tasks. For their practical application at the workplace, it is also important that their applicability is examined not only in the laboratory but also under more realistic conditions. This becomes particularly important when considering the technological advancements regarding mobile EEG technology that have simplified signal registration outside of shielded rooms (Mihajlovic et al., 2015; Aricò et al., 2018; Radüntz, 2018; Baek et al., 2019; Radüntz and Meffert, 2019).

In our prior work we developed a mental-workload classifier that does not need retraining, neither for new subjects nor for new tasks (Radüntz, 2017). In a laboratory study conducted with 54 subjects and during execution of well-established cognitive tasks, we developed the so-called Dual Frequency Head Maps (DFHM). These head maps consist of personalized spectral features and their spatial occurrence (i.e., frontal theta-band and parietal alpha-band powers). Support vector machines are used for classification in three classes: low, moderate, or high workload. Under laboratory conditions, we successfully proved the DFHM method as universally applicable with fixed parameters for mental-workload indexing. For proofing the reliability and reproducibility of our DFHM method's results under realistic conditions, we conducted a study in cooperation with the German Aerospace Center and focused on air traffic controllers. The following four research hypotheses were formulated for the DFHM-validation study:

1. The DFHM method yields stable results under similar task-load conditions independently of the time of measurement.
2. The DFHM method is able to assess workload differences that arise from different traffic-volumes conditions.
3. The DFHM method is able to assess workload differences that arise from an exceptional-event condition.
4. The objectively measured workload assessed by the DFHM method is related to controller's subjectively experienced workload.

## 2. Materials and Methods

### 2.1. Research Design

Our study took place at the Air Traffic Management and Operations Simulator (ATMOS) of the German Aerospace Center (DLR) in Braunschweig. Thereby, air traffic controllers focused on simulated arrival management procedures presented on the monitor and interacted along the experimental task with pseudo pilots who simulated the cockpit crews. The implemented simulation scenarios differed regarding two factors that were responsible for mental workload variations of air traffic controllers as suggested by Averty et al. (2004). The first one was the traffic load. In our case, we had four levels corresponding to four different numbers of aircraft per hour (ac/h). The second factor was an exceptional event that could occur or not. This event was a pilot's request for a flight prioritization because of a sick passenger on board. The priority-flight request could occur around the 11th min of the 20–25 min lasting scenario. Both factors led to the eight scenarios presented in Table 1.

**Table 1:** Independent variables and simulation scenarios.

		**Number of aircraft per hour**			
		**Low (25 ac/h)**	**Medium (35 ac/h)**	**High (45 ac/h)**	**Very high (55 ac/h)**
Exceptional event	No	Scenario 1	Scenario 3	Scenario 5	Scenario 7
	Yes	Scenario 2	Scenario 4	Scenario 6	Scenario 8

### 2.2. Procedure and Subjects

We asked subjects to participate in a 2-days experiment where they had to complete the above-mentioned eight traffic scenarios in randomized order. The experimental procedure is outlined in Table 2. The investigation consisted of an introductory session and the main experiment. During the introductory session participants completed demographic questionnaires, were briefed regarding the research goals and experimental procedure of the following 2 days, and had a training session at the simulator in order to get familiarized with the environment. During the main experiment the subjects completed four of the simulation scenarios while the remaining four were conducted on the second day.

**Table 2:** Experimental procedure.

**Duration (min)**	**Procedure**	
	**Day 1: ca. 12.30–17.30**	**Day 2: ca. 9.30–12.30**
120	Briefing, training	
65	Two simulation scenarios	Two simulation scenarios
15	Break	Break
65	Two simulation scenarios	Two simulation scenarios

In our study, we examined 21 subjects in the age between 22 and 64 years (2 females, 19 males, mean age 38 ± 11). Subjects were from different airports, had different work experience, revealed different work positions (i.e., 13 approach controllers, three tower controllers, and five employees of the DLR), and had experienced different work demands. However, all of them had adequate expertise to handle the arrival management simulation.

All of the investigations acquired were approved by the local review board of our institution and complied with the tenets of the Declaration of Helsinki. All procedures were carried out with the adequate understanding and written consent of the subjects.

### 2.3. Subjective Ratings

In order to register the subjectively experienced workload, we used the instantaneous self-assessment (ISA) questionnaire. This was developed for the assessment of air traffic controller's mental workload (Brennan, 1992; Jordan, 1992; Kirwan et al., 1997) and consisted of a one-dimensional scale. Thus, it was quickly and easily conducted in an interval of 5 min during all eight scenarios. According to their feeling during the previous 5 min, subjects indicated their workload using a touch screen. Thereby, they selected one of the following five values: (1) under-utilized, (2) relaxed, (3) comfortable, (4) high, and (5) excessive.

Analysis of the ISA questionnaire results was particularly relevant for our fourth hypothesis related to controller's subjectively experienced workload. Based on these we developed a so-called workload-sensitivity index that considered the individual range of experienced workload during different task-load conditions.

Subject's normalized workload-sensitivity index s_a_ was based on a linear model for the dependence of subjectively experienced workload as assessed by the ISA questionnaire and traffic load. In Fürstenau et al. (2020), we showed that the linear model was able to predict the ISA value with a high confidence for means across the subjects and provided reasonable linear correlation coefficients for the individuals. Independence from the arbitrary ISA values was achieved via normalization by scales' means, i.e., (traffic load_max_ + traffic load_min_) / 2 for the traffic volume and (ISA_max_ + ISA_min_) / 2 for the subjective workload, resulting in anticorrelated (normalized) sensitivity and intercept s_b_ = 1 − s_a_. ISA-scale means were conducted individually for each subject based on the ISA-extreme values from their regression lines.

Our workload-sensitivity index ranged between 0.32 and 1.23, and was used for subject clustering. The aim of this clustering was an improved investigation of the cognitive phenomena only of those subjects that actually experienced different workload levels. Subjects with an index below the median of 0.8 were clustered as not sensitive, while subjects with an index equal or above the median as workload sensitive. Generally speaking, workload-sensitive subjects experienced more workload variation during the different simulation scenarios whereas the not-sensitive subjects rated the subjectively experienced workload with less variation.

### 2.4. EEG and DFHM-Workload Index

Biosignal processing and all calculations were done with MATLAB.

**Figure 1:**
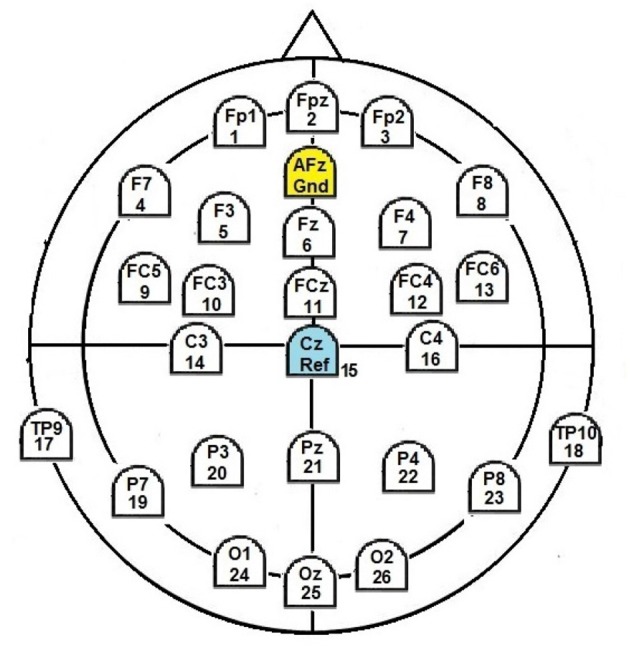
EEG layout used.

For EEG registration we used g.tec's g.LADYbird/g.Nautilus system with 25 active electrodes placed at positions according to the 10–20-system (Figure 1). Registration was carried out with a sample rate of 500 Hz and with reference to electrode Cz. For signal recording we used g.tec's Matlab interface.

After recording, the EEG was filtered with a bandpass filter (order 100) between 0.5 and 40 Hz for enhancing the separation accuracy of the following analysis for artifact rejection (Fernandez, 2009; Omatu et al., 2010; Pignat et al., 2013; Winkler et al., 2015). Independent component analysis [ICA, Infomax algorithm (Makeig et al., 1996)] for artifact rejection was applied to the signal. Components to reject were manually selected (i.e., on average 16 out of 25 per subject). In order to increase topographical localization, we applied a simple Hjorth-style surface laplacian filter using eight neighbors (Hjorth, 1975). This spatial high-pass filter was aimed to attenuate large-scale scalp signals and amplify localized signals.

The artifact-free EEG was transformed to average reference and cut into segments of 1 s length, overlapping by 0.5 s. By means of Fast Fourier Transformation (FFT) we computed the workload relevant frequency bands (theta: 4–8 Hz, alpha: 8–12 Hz) over the segments. Table 3 shows the general tendencies of both frequency bands exemplary for two electrodes. Involvement of all electrodes, the combination of both frequency bands, and the personalization of the band-power values aim at enhancing workload classification and constitute the DFHM that were generated as outlined in Radüntz (2017). In brief, we applied a theta-bandpass filter to the signals of the frontal electrodes and an alpha-bandpass filter to the signals of the parietal electrodes and calculated for each participant, each electrode, and each segment the z-scores of theta and alpha band power. The individual mean and standard deviation for z-score calculation were obtained from subject's segments of the first minute of each scenario. This compilation of the z-scores of the theta band power from the frontal electrodes and alpha band power from the parietal electrodes constituted the DFHM for each EEG segment. Next, each DFHM from the simulation scenarios' segments was classified using the already trained SVM classifier from the laboratory study. Retraining of the DFHM classifier was not necessary neither for the new subjects nor for the new tasks. The general characteristic of these maps and thus, the classifier is universally applicable because of the z-score calculation. For more information about the DFHM and classifier development, we refer the interested reader to our method article (Radüntz, 2017).

**Table 3:** Mean and standard deviation (in parenthesis) of the α and θ frequency band powers exemplary for two electrodes averaged over the subjects for each simulation scenario.

**Number of aircraft per hour**	**25 ac/h**	**35 ac/h**	**45 ac/h**	**55 ac/h**
**Without an exceptional event**				
θ frequency band power (Fz electrode)	16.5 (4.0)	17.5 (4.3)	17.4 (3.7)	18.2 (3.8)
α frequency band power (Pz electrode)	26.1 (6.1)	25.2 (5.4)	25.4 (5.7)	25.1 (5.2)
**With an exceptional event**				
θ frequency band power (Fz electrode)	16.7 (3.7)	17.4 (4.1)	17.3 (3.7)	17.8 (3.9)
α frequency band power (Pz electrode)	26.0 (6.0)	25.5 (5.6)	25.0 (5.2)	24.9 (4.4)

We obtained every 0.5 s a value of 1 (low workload), 2 (moderate workload), or 3 (high workload). We applied a moving-average time window of 30 s as suggested by Abbass et al. (2014a) and adjusted the result in order to gain a DFHM-workload index between 0 and 100 (Equation 1; with *t*: workload index at time *t*, DFHM (i): classification value of DFHM from segment *i*).

(1) $$\begin{array}{l} \text{WKLindex}\left(\text{t}\right)=\left(\overset{t}{\underset{i=t-59}{\sum }}DFHM\left(i\right)-60\right)/120*100 \end{array}$$

In particular, for each moving-average time window of 30 s we firstly calculated the sum of the 60 values resulting from the DFHM every 0.5 s. In order to have a baseline of 0, we subtracted the minimum-possible sum of 60 for the case where all DFHM of the window indicated a low workload of 1. Thus, the maximum-possible sum for the case where all DFHM of the window indicated a high workload was 120. Dividing by the latter and multiplying by 100 provided the percentage amount of high-workload segments in a time-window of 30 s. This constituted the DFHM-workload index between 0 and 100 computed every 0.5 s.

### 2.5. Statistical Analysis

For evaluating our first hypothesis and proof the reliability of the DFHM index, we calculated the DFHM-index average over the first 5 min of each simulation and correlated the means of scenarios with same traffic load.

For investigating the ability of the DFHM method to assess mental workload arising from the traffic volume (hypothesis 2) and the occurrence of an exceptional event (hypothesis 3), we looked at the time slots immediately after the time of a possible priority-flight request. This was triggered in the data using g.tec's g.TRIGbox. For scenarios with a priority-flight request we considered a DFHM-index segment of 2.5 min starting from the request time point. For scenarios without a priority-flight request we used the same time slots. We carried out an analysis of variance (ANOVA) with the slots' mean DFHM index as dependent variable. We utilized a repeated-measures design with two within-subject factors (two levels for the priority-flight request factor and four levels for the traffic-volume factor). General differences between the levels were examined and tested with a *post-hoc* test (Bonferroni corrected).

Finally, we addressed the issue of DFHM-index workload registration in relation to subjects' subjectively experienced workload (hypothesis 4). We clustered our subjects in two groups using the median of our workload-sensitivity index that was calculated from the ISA ratings. This yielded nine subjects that subjectively did not experience workload variations between the scenarios and 12 workload-sensitive subjects. We carried out a mixed ANOVA with cluster affiliation as between-subject factor followed by a two-factorial ANOVA for each cluster separately for determining the simple main effects of our factors. The dependent variable, within-subject factors, and levels were identical with those mentioned above. Similarly, we utilized a repeated-measures design and examined the differences with *post-hoc* tests (Bonferroni).

Statistical calculations were conducted using SPSS and the significance threshold was set at 5%.

## 3. Results

### 3.1. DFHM Index Under Similar Conditions

Our first hypothesis was concerned with the ability of the DFHM method to yield stable results under similar task-load conditions. Scenarios with and without priority-flight request were identical regarding their traffic volumes until the 10th min where the request could occur. Thus, we decided to use only the first 5 min of each simulation for assuring similar task load conditions between both values to be correlated. By taking a larger slot, the scenarios would increasingly differ the more time passed away as consequence of the interactive communication of the ATC with the pseudo pilots.

Correlation analyses between the mean DFHM index of the first 5 min of simulation scenarios with same traffic load showed significant positive correlations. These were particularly high for the traffic-load conditions of 35 and 45 ac/h and less pronounced for the lowest traffic load of 25 ac/h. Person's correlation coefficients are presented in Table 4.

**Table 4:** Correlation analysis of DFHM-index means over the time slot 0–5 min during scenarios with equal traffic-load volume (*N* = 21, ***p ≤ 0.001).

	**Traffic load**			
	**25 ac/h**	**35 ac/h**	**45 ac/h**	**55 ac/h**
Pearson's correlation coefficient	0.671***	0.809***	0.798***	0.746***

### 3.2. DFHM Index Related to Traffic Load and Priority-Flight Request

In order to evaluate the ability of the DFHM method to assess workload differences arising from different traffic-volume and exceptional-event conditions, we considered the results of the ANOVA. They were calculated with the two within-subject factors traffic-load and priority-flight request. The results are summarized in Table 5.

**Table 5:** Analysis of DFHM index across simulation conditions over all subjects and subjects' clusters, respectively.

		***F***	***p***	**η^2^**
Traffic load	All	22.953^*a*^	0.001	0.534
	Workload-sensitive subjects	36.815	0.001	0.769
	Not-sensitive subjects	2.762	0.064	0.257
Priority-flight request	All	1.349	0.259	0.063
	Workload-sensitive subjects	15.636	0.002	0.587
	Not-sensitive subjects	1.311	0.285	0.141
Traffic load and	All	0.214	0.886	0.011
priority-flight request	Workload-sensitive subjects	0.936	0.434	0.078
	Not-sensitive subjects	0.440	0.726	0.052

*Values of 0.001 are actually p ≤ 0.001*.

a *Indicates Mauchly's test of sphericity was significant (p < 0.05) and a Greenhouse-Geisser correction was made to degrees of freedom*.

**Figure 2:**
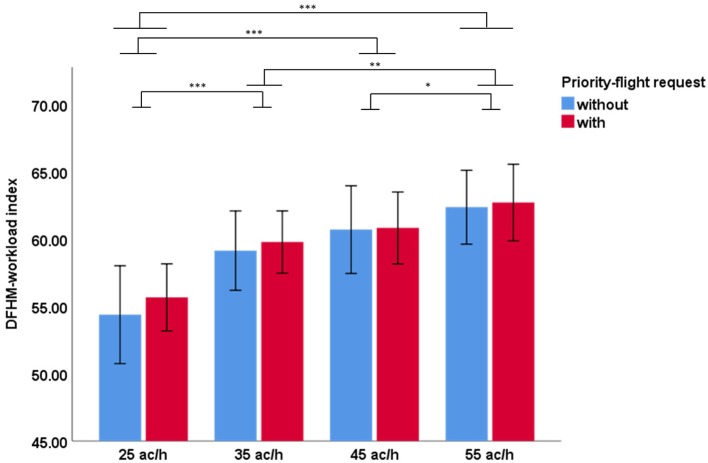
Mean DFHM index over 21 participants measured during the 2.5 min slots after a possible priority-flight request across simulation conditions with (red) and without (blue) priority-flight request at different traffic loads (Bonferroni corrected *post-hoc* tests: ***p ≤ 0.001; **0.001 < *p* ≤ 0.01; *0.01 < *p* ≤ 0.05; error bars indicate 95% confidence interval).

Related to our second hypothesis the traffic load had a significant main effect on the workload as assessed by the DFHM index. Bonferroni corrected *post-hoc* tests showed significant differences between all levels except between the 35 and 45 ac/h conditions. The DFHM-workload index increased with increased traffic. Figure 2 shows the results. The impact of the priority-flight request as related to our third hypothesis did not became significant. No interaction effect could be obtained between traffic load and priority-flight request.

**Figure 3:**
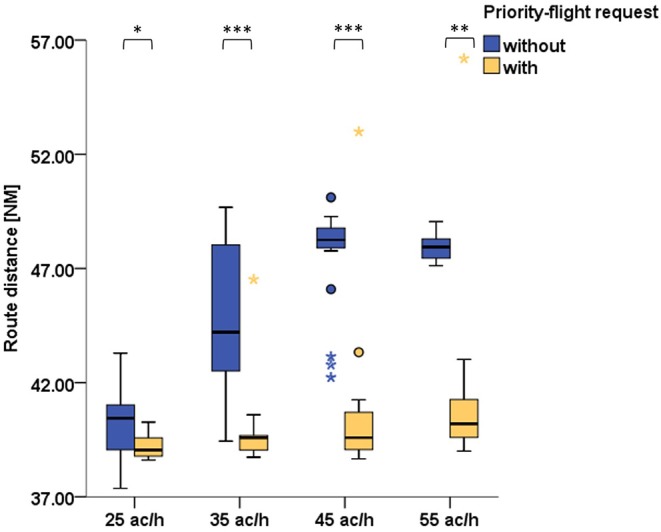
Comparison of prioritized aircraft's route distance during scenarios with priority-flight request (orange) and during scenarios with same traffic volume but without prioritization (blue) for all 21 subjects (Wilcoxon signed-ranks tests with Bonferroni correction: ***p ≤ 0.001; **0.001 < *p* ≤ 0.01; *0.01 < *p* ≤ 0.05).

For assuring that air traffic controllers indeed prioritized the aircraft, we evaluated the route distances of the same aircraft with and without priority-flight request. In both cases the route distance taken was the length of trajectory between the initial contact time point and landing. A shorter route distance for the requesting aircraft indicated that air traffic controllers complied with the priority-request condition (Figure 3). Wilcoxon signed-ranks tests (with Bonferroni correction) indicated that the route distance was significantly shorter during scenarios with priority-flight request compared to scenarios with same traffic volume but without priority-flight request (Table 6).

**Table 6:** Wilcoxon signed-ranks tests (with Bonferroni correction) for comparison of prioritized aircraft's route distance during scenarios with priority-flight request and aircraft's route distance during scenarios with same traffic volume but without priority-flight request.

	**Median (range) route distance [NM]**		***Z***	***p***	***r***
	**Without priority-flight request**	**With priority-flight request**			
**All subjects (*****N*** **=** **21)**					
25 ac/h	40.44 (5.92)	39.05 (1.66)	−2.52	0.047	−0.55
35 ac/h	44.21 (10.24)	39.58 (7.79)	−4.02	0.001	−0.88
45 ac/h	48.25 (7.88)	39.59 (14.33)	−3.84	0.001	−0.84
55 ac/h	47.94 (1.93)	40.20 (17.19)	−3.46	0.002	−0.76
**Workload-sensitive subjects (*****N*** **= 12)**					
25 ac/h	40.37 (4.83)	38.95 (1.10)	−1.49	0.544	−0.43
35 ac/h	44.18 (9.93)	39.36 (1.14)	−3.06	0.009	−0.88
45 ac/h	48.03 (7.04)	39.61 (14.33)	−2.67	0.031	−0.77
55 ac/h	47.61 (1.93)	40.42 (17.19)	−2.35	0.074	−0.68
**Not-sensitive subjects (*****N*** **=** **9)**					
25 ac/h	40.45 (4.68)	39.52 (1.64)	−1.96	0.203	−0.65
35 ac/h	44.64 (7.17)	39.59 (7.74)	−2.67	0.031	−0.89
45 ac/h	48.74 (2.07)	39.58 (1.85)	−2.67	0.031	−0.89
55 ac/h	48.13 (1.16)	39.91 (3.97)	−2.67	0.031	−0.89

*Values of 0.001 are actually *p* ≤ 0.001*.

### 3.3. DFHM Index Related to Subjectively Experienced Workload Variations

For our last hypothesis, results from the mixed ANOVA showed statistically significant interaction effects between cluster affiliation and traffic load [*F*_(3, 57)_ = 7.215, *p* < 0.001, η^2^ = 0.275] as well as between cluster affiliation and priority-flight request [*F*_(1, 19)_ = 9.517, *p* = 0.006, η^2^ = 0.334]. No significant interaction effect could be obtained between all three factors cluster affiliation, traffic load, and priority-flight request [*F*_(3, 57)_ = 1.195, *p* = 0.319, η^2^ = 0.059].

In the following, we analyzed the DFHM index for the workload-sensitive cluster and the not-sensitive cluster separately. For the workload-sensitive cluster the ANOVA yielded a significant main effect for the traffic load and priority-flight request. Bonferroni corrected *post-hoc* tests showed significant differences between all traffic-load levels except between the highest traffic load volumes with 45 and 55 ac/h. The DFHM-workload index increased with increased traffic load and was higher during scenarios with priority-flight request. No interaction effect could be obtained between both factors.

**Figure 4:**
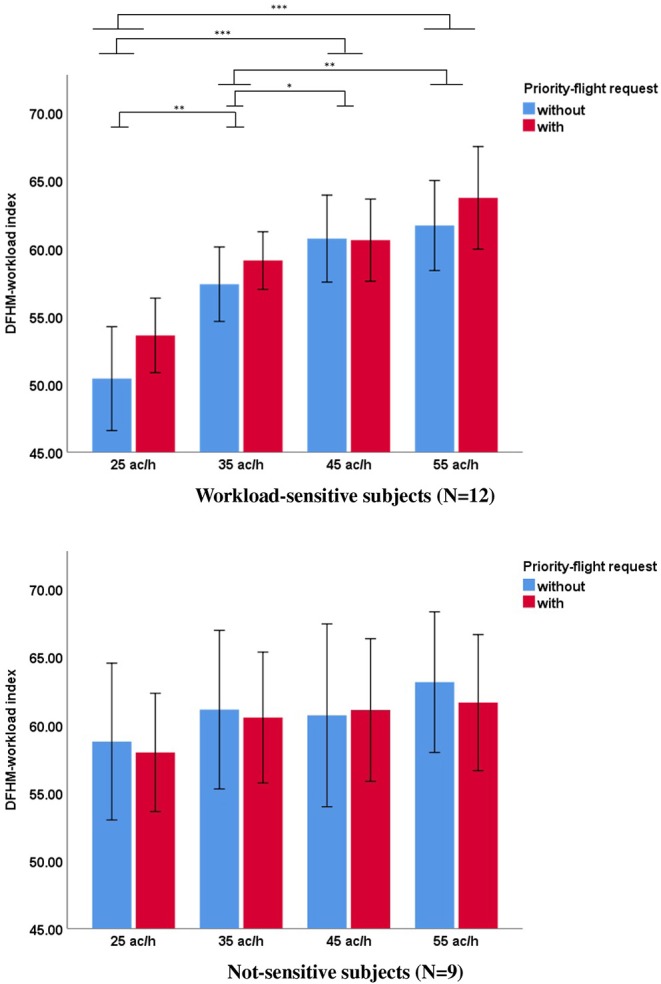
Mean DFHM index during scenarios with (red) and without (blue) priority-flight request at different traffic loads for workload-sensitive (top row) and not-sensitive (bottom row) subjects (Bonferroni corrected *post-hoc* tests: ***p ≤ 0.001; **0.001 < *p* ≤ 0.01; *0.01 < *p* ≤ 0.05; error bars indicate 95% confidence interval).

For the not-sensitive cluster no significant differences could be obtained for none of the factors. The results are summarized in Table 5 and shown in Figure 4.

### 3.4. Performance Related to Subjectively Experienced Workload Variations

**Figure 5:**
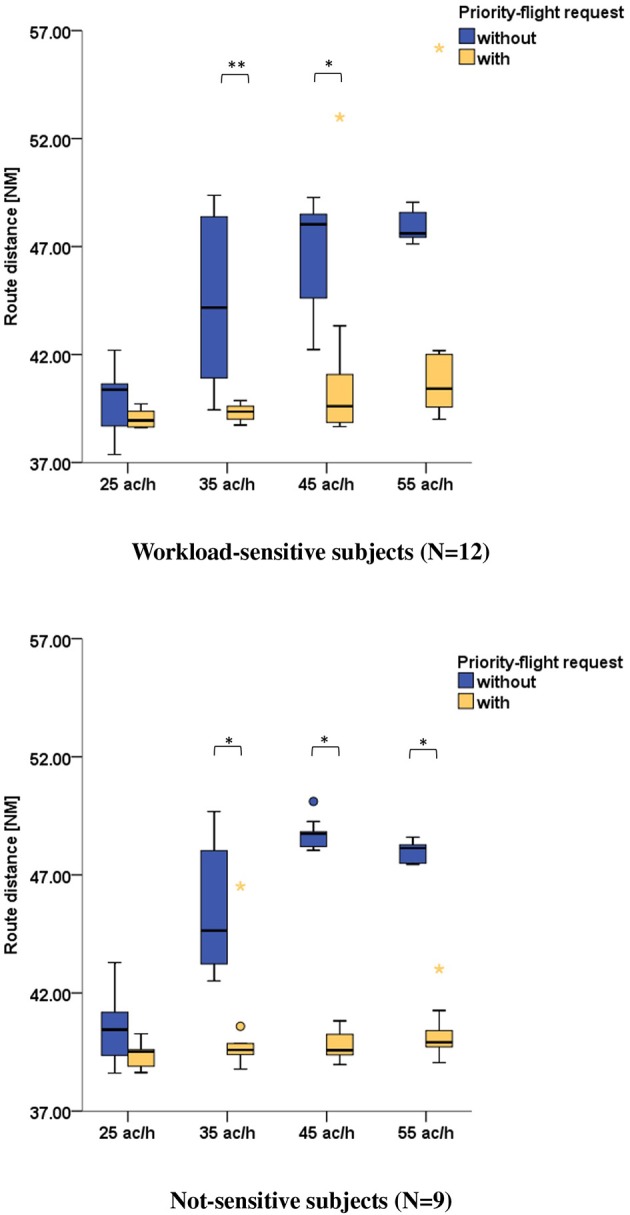
Comparison of prioritized aircraft's route distance during scenarios with priority-flight request (orange) and during scenarios with same traffic volume but without prioritization (blue) for workload-sensitive (top row) and not-sensitive (bottom row) subjects (Wilcoxon signed-ranks tests with Bonferroni correction: **0.001 < *p* ≤ 0.01; *0.01 < *p* ≤ 0.05).

In addition to the DFHM index we evaluated the performance of the air traffic controllers for the workload-sensitive and not-sensitive clusters. As measure of performance we employed the route distances and loss of separation. Evaluation of route distance between aircraft with priority-flight request and without was conducted separately for each cluster. The results are presented in Table 6 and Figure 5 and revealed similar tendencies for both clusters, i.e., the route distance of the requesting aircraft was significantly shorter during 35 and 45 ac/h traffic load. During the 55 ac/h condition this held true only for the not-sensitive cluster. No significant difference could be found for none of the clusters during the 25 ac/h condition.

**Figure 6:**
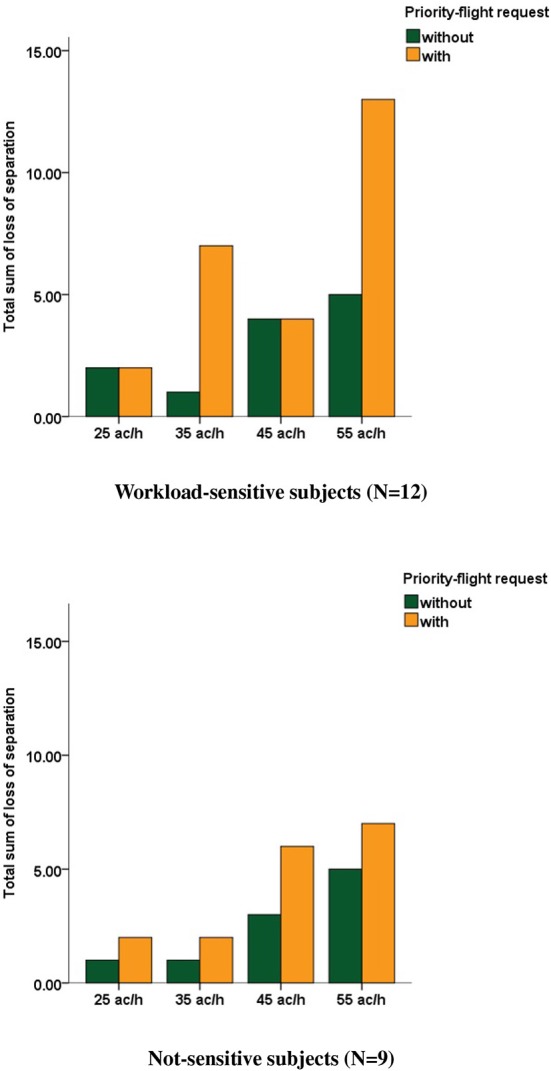
Total sum of loss of separation computed over all workload-sensitive (top row) and not-sensitive (bottom row) subjects during scenarios with (orange) and without (green) priority-flight request at different traffic loads.

Evaluation of loss of separation between aircraft was conducted according to the minimum separation standards specified by the authorities and based on the standards of the International Civil Aviation Organization (2011). The separation minima were breached when lateral distance between two aircraft was smaller than the required vake vortex separation, i.e., 3 NM (nautical miles) for two medium type aircraft and 5 NM for a medium aircraft following a heavy aircraft, and simultaneously vertical distance between these aircraft was smaller than 1,000 ft. In general, the number of loss of separation was low (i.e., around zero) and thus not appropriate for statistical evaluation. For the sake of completeness, Figure 6 illustrates the results for each cluster separately.

## 4. Discussion

In our study, we aimed in validating our method for mental workload registration by means of DFHM. The method was already proofed in a laboratory setting but further evaluation was needed. Our current validation study was conducted under realistic conditions, with real tasks, and new subjects, i.e., in an air traffic control simulator, with arrival-management tasks, and air traffic controllers. Our sample set consisted of 21 subjects that completed eight simulation scenarios in randomized order. The simulation scenarios differed regarding their traffic load that consisted of four levels and a priority-flight request that could occur around the 11th min of simulation or not. We registered the EEG during the simulations and computed the DFHM-workload index for each subject and scenario. We did not retrain the classifiers neither for the new tasks nor for the new subjects. The gained results were promising.

The DFHM index gave stable results with highly significant correlations between scenarios with similar traffic-load conditions as stated by hypothesis 1. We observed that these correlations were particularly pronounced during the medium and high traffic volumes and less strong for the low-traffic volume. During the latter, requirements were very low and allowed air traffic controllers to have task-unrelated thoughts in order to cope with boredom (Cummings et al., 2015). Boredom proneness, coping strategy as well as the kind of task-unrelated thoughts could have mitigated the correlation between the two 25 ac/h scenarios. One could argue that there might be also other factors that might influence results stability across scenarios, e.g., effects of learning and fatigue in the course of time, the interaction with the pseudo pilots, or the initial excitement during the presentation of the first scenario. However, our sample was very specialized. Air traffic controllers are highly trained and it seemed unlikely that they gained knowledge in the course of the experiment. The initial training phase prior to our experiment was aimed to familiarize the subjects with the environmental conditions and eliminate issues related to these. For minimizing fatigue effects, we followed the regulations of working-time organization for air traffic controllers that prescribe a break after 120 min of work. Each scenario had a maximal duration of 25 min, a break took place after two scenarios (i.e., after 50 min), and the daily session consisted of four simulation scenarios. Effective daily-work time was 100 min the most. Hence, fatigue effects should be minimal. Presentation order of the scenarios was randomized and should compensate the initial excitement across subjects. Finally, air traffic controllers should be used to the interaction with different pilots from their daily work experience. Hence, we concluded that workload differences should result from the experimental conditions and the DFHM-workload index should be comparable during the first minutes of simulations with equal traffic load. Nevertheless, we have to draw attention on the increased requirement on our DFHM-workload index because of our 2-days experiment with randomized presentation order of the scenarios. Keeping this in mind, results from the correlation analysis appear encouraging.

While the first hypothesis was concerned with test-retest reliability, the second and third hypotheses addressed the issue of validity of the DFHM method as workload indexing technique. The DFHM index was able to assess significant differences between the different levels of air traffic volume as stated by hypothesis 2. Problematic were the neighboring levels with 35 and 45 ac/h that could not be significantly discriminated by the DFHM-workload index when considered over all subjects. The same held true regarding the priority-flight request although evaluation of the route distance of the requesting aircraft indicated that air traffic controllers complied with the task. At this stage hypothesis 3 had to be rejected when considered over all subjects.

More insight regarding intra-individual differences linked to the DFHM-workload index was gained from subject clustering by means of the subjectively experienced workload differences during the scenarios. Thus, our fourth hypothesis dealt not only with issues of validity but also of consistency between subjective and objective measuring methods. We were able to obtain highly significant interaction effects between subjective workload-cluster affiliation and traffic load as well as priority-flight request. For subjects reporting that they experienced workload variation between the different scenarios, the DFHM-workload index yielded significant differences between traffic-load levels and priority-flight request conditions. Interestingly, for these subjects the DFHM index was able to differentiate between the neighboring levels with 35 and 45 ac/h but not between the 45 and 55 ac/h conditions. Descriptive evaluation of Figure 4 indicates that for the workload-sensitive subjects there was a ceiling effect regarding traffic volume. This occurred for traffic-volumes >45 ac/h and seemed reasonable when taken into account that a traffic volume of 55 ac/h was a condition that is highly improbable in reality for single-runway operations. Latter was constructed for the simulation in order to create an extreme situation that would definitely challenge the operators and increase their workload. Nevertheless, air traffic controllers are trained to adjust their work strategies in order to assure safety. This strategy change could be a reason for the ceiling effect during the very high traffic-load condition. However, the occurrence of a priority-flight request during the very high traffic-load condition led to a further increase of the DFHM-workload index. Unfortunately, our small sample size and the even smaller amount of subjects in the clusters did not allow for elaborated statistics regarding interaction effects.

In contrast to the significant differences obtained for the workload-sensitive cluster, the DFHM-workload index behaved differently for the not-sensitive cluster and did not yield any significant differences for any of the factors. In our opinion, this fit well to our fourth hypothesis and indicated that the objectively measured workload assessed by the DFHM method corresponded to controller's subjectively experienced workload. To sum up, hypothesis 2 and 3 proofed true only for subjects that experienced workload differences also subjectively during the scenarios. The workload insensitivity of subjects might appear odd when considering the high variability of our experimental design. An explanation might be traced back to the different cognitive strategies in task solving, both intra- and inter-individually, that might influence the experienced workload. Each controller had a different way to handle the traffic. This was possibly related to the different individual experience level from daily-work life as linked to the size of the airport he was working, the different ages, but also personality traits. Unfortunately, we were not able to identify personal characteristics for each cluster that might be responsible for the different perceptions of workload. More research is needed in order to understand which individual factors contribute to these interpersonal differences.

Analyses of performance data emphasized these findings. Results revealed a tendency to more loss of separation and lower prioritization during the extreme traffic load condition for workload-sensitive subjects that was less pronounced for the not-sensitive subjects. These might be an additional indicator that subjects from the workload-sensitive cluster experienced more workload compared to the others as evident by the DFHM-workload index. As a side note, readers might wonder that route-distance difference was low between the 25 ac/h scenarios with and without priority-flight request. This was reasonable because of the low-traffic volume that allowed air traffic controllers to instruct pilots to fly direct routes to the final approach even without a priority-flight request by the pilot. Conversely, a weaker significance level for the route-distance difference between both 55 ac/h scenarios could be linked to a smaller ability to prioritize the aircraft due to increased demands resulting from the high-traffic load.

A limitation of our study was the realization of the exceptional event as recurring priority-flight request. The surprising effect of the unexpected event might have diminished after the first occurrence of the request. Thereafter, air traffic controllers might have adjusted their strategy and behavior in order to be prepared to appropriately react to a recurring event. Studies that aim to understand the effect of an unexpected event on workload, should pay more attention on this issue. Finally, a larger sample size would be beneficial.

## 5. Conclusions

With the development and availability of low-cost and easy-to-use EEG sensors, amplifiers, and signal-processing algorithms over the last 20 years (Lopez-Gordo et al., 2014; Radüntz, 2018; Flumeri et al., 2019; Radüntz and Meffert, 2019), certain frequency bands of the EEG have proven to be particularly informative and were therefore being used more and more frequently for mental-workload detection. The numerous studies published after the year 2000 were fairly different, depending on the specific question, purpose, and expertise of the authors (Lin et al., 2006; Berka et al., 2007; Kohlmorgen et al., 2007; Borghini et al., 2014; Ke et al., 2014; Bashivan et al., 2015; Aricò et al., 2016). Initially, the spectral power in the alpha and theta frequency bands were identified as particular relevant, analyzed, and tested variance-analytically related to mental workload. In the last few years classifiers that relied on large property vectors of EEG activity were increasingly developed. Thereby, the derived parameters let barely identify the concrete psycho-physiological meaning of the EEG activity. We aimed to avoid this issue by making use of well-established parameters that should be valid for different subjects and tasks.

In our article, we particularly addressed questions of functionality outside the laboratory, stability of results, and the generalization properties of the DFHM-workload index, inter-individually and cross-task. In conclusion, it can be stated that a reliable determination of mental workload in a realistic setting and with real-world scenarios was possible. Continuous determination under real conditions, however, requires further systematic investigations. Although the temporal resolution of the EEG permits a workload determination in the range of seconds, the states to be detected originate from long-running procedures and therefore require further research about an informative time frame for averaging classifier's output. Future promising applications of the DFHM-workload index include research about effects of human-computer interaction, human factors, ergonomic designs of the cognitive state as an objective method for development and testing new interfaces, determination of the effectiveness of training and simulation programs, or even the characterization of group dynamics when collecting synchronous EEG data from multiple subjects. The recently increasing attempts of a real-time application of EEG parameters to determine vigilance, emotion, workload, and stress are accompanied by the effort of catchy visualization of the results. With an easy accessibility of such systems, however, there is also an increasing risk of uncritical assessment and interpretation of the measured values by laymen.

## Data Availability Statement

The conducted data used to support the findings of this study are restricted by the ethics committee of the Federal Institute for Occupational Safety and Health in order to protect subjects privacy according to data-protection regulations. Data can be made available from the corresponding author upon request and after approval of the legal department for researchers who meet the criteria for access to confidential data.

## Ethics Statement

The studies involving human participants were reviewed and approved by Federal Institute for Occupational Safety and Health. The patients/participants provided their written informed consent to participate in this study.

## Author Contributions

TR initiated the project and was responsible for the overall conception of the investigation. TR, TM, and NF developed the research design of the study. TM was responsible for the implementation of the simulation scenarios and the overall technical support. TR was responsible for the signal processing, data analysis, and method of evaluation. The study was supervised by TR. Data interpretation was performed by TR and BM. The manuscript was written by TR. Final critical editing was performed by TM, NF, and BM.

## Conflict of Interest

The authors declare that the research was conducted in the absence of any commercial or financial relationships that could be construed as a potential conflict of interest.
